# South African Flag Sign in Left Anterior Descending Artery Occlusion Revealing Multivessel Coronary Artery Disease

**DOI:** 10.7759/cureus.101183

**Published:** 2026-01-09

**Authors:** Lucio Giuseppe Granata, Emanuele Grasso, Marcello Marchetta, Francesco Amico

**Affiliations:** 1 Cardiology, Cannizzaro Emergency Hospital, Catania, ITA; 2 Cardiology, San Vincenzo Hospital, Taormina, ITA; 3 Cardiology, Hospital Tor Vergata Roma, Rome, ITA

**Keywords:** ecg criteria, first diagonal artery, heart failure with reduced ejection fraction, high lateral stemi, ischaemic heart disease, left anterior descending coronary artery (lad), occlusion myocardial infarction, omi, south africa flag sign, st-elevation myocardial infarction (stemi)

## Abstract

Early recognition of subtle or atypical electrocardiographic patterns of acute coronary occlusion remains a major challenge in contemporary practice. The "South African flag sign" (SAFS) is characterised by concomitant ST-segment elevation in leads I-aVL and in the non-contiguous lead V2, together with reciprocal ST-segment depression in lead III and often in the remaining inferior leads, resulting in a distinctive spatial distribution of injury currents in the 4x3 electrocardiogram format, reminiscent of the South African national flag. It represents a marker of mid-anterior/anterolateral transmural ischaemia due to acute occlusion of the first diagonal (D1) branch of the left anterior descending (LAD) coronary artery. However, this pattern is not currently incorporated into international guidelines and remains underrecognised in clinical practice.

We report the case of an elderly woman presenting with acute coronary syndrome (ACS) and transient ST-segment elevation, in whom the electrocardiogram revealed the SAFS. Urgent coronary angiography demonstrated a subocclusive proximal LAD bifurcation lesion extending to D1, as well as a critical mid-segment stenosis of the right coronary artery, an additional finding not previously described in patients presenting with this electrocardiographic sign. In addition, an intermediate branch free of critical disease and a well-developed second diagonal branch were identified, the latter showing flow limitation secondary to the proximal LAD bifurcation stenosis. Both critical lesions were treated with percutaneous coronary intervention and stent implantation. Despite reperfusion, the patient developed moderate left ventricular dysfunction. Her subsequent clinical course was uneventful, and she was discharged 10 days later.

This case underscores the diagnostic and clinical relevance of SAFS in the setting of ACS, including its persistence despite the absence of conventional ST-elevation myocardial infarction (STEMI) criteria, and highlights the need for increased awareness of this electrocardiographic manifestation.

## Introduction

Prompt recognition of acute coronary occlusion (ACO) remains a cornerstone of early management in patients presenting with suspected acute coronary syndrome (ACS). While classical ST-segment elevation (STE) patterns allow rapid identification of transmural ischemia in many cases, a significant proportion of total or near-total ACO present with more nuanced or unconventional electrocardiographic findings. These “ST-segment elevation myocardial infarction (STEMI)-equivalent," or more properly occlusive myocardial infarction (OMI) patterns, now increasingly acknowledged in the literature, highlight the limitations of the STEMI paradigm relying solely on traditional territorial ST-elevation criteria and underscore the need for heightened diagnostic vigilance [1].

Mid-anterior/anterolateral myocardial infarction represents one of the territories in which ischemic changes may be particularly subtle. The anterolateral wall, supplied variably by the first diagonal branch, obtuse marginal branches, or, less commonly, by the ramus intermedius, often generates electrocardiographic patterns that do not conform to the more familiar inferior or anterior ischemic signatures. ST-segment deviations may be of low amplitude, appear in non-contiguous leads as defined by current guidelines, or even be interpreted as non-ST-segment elevation myocardial infarction (NSTEMI). As a result, acute occlusion of these vessels can be easily missed, contributing to delays in reperfusion and potentially worse clinical outcomes [2].

Within this context, the South African flag sign (SAFS) has emerged as a proposed ECG pattern indicative of transmural ischemia. It is defined by simultaneous STE in leads I-aVL and non-contiguous lead V2, accompanied by reciprocal depression in lead III and often with other inferior leads. The orientation of these changes reflects an injury vector directed superiorly and laterally, usually consistent with specific occlusion of the first diagonal branch (D1) of the left anterior descending (LAD) coronary artery.

Although the original pattern was first described in 1994 by Sclarovsky et al. in 1.7% of 471 patients with anterior myocardial infarction [3], the visual configuration was proposed for educational purposes by Littmann only in 2016. This provided a visually striking and physiologically coherent marker that may alert clinicians to an occlusive process not captured by classical STEMI criteria. It resembles the green shape of the South African national flag when the electrocardiogram (ECG) is displayed in the 4×3 format [4].

Despite its potential diagnostic value, SAFS remains absent from current European and American guideline frameworks and is therefore not widely recognised in emergency settings. This gap may hinder timely reperfusion in patients with mid-anterior/anterolateral OMI, a subgroup already at risk of underdiagnosis due to the subtlety of ECG manifestations. Greater awareness of SAFS could help bridge an important blind spot in the early assessment of ACS and reduce reliance on patterns that prioritise more common infarction territories.

In this case report, we describe a patient in whom the presence of SAFS on the initial ECG served as an important clue to an underlying ACO. Through this case, we aim to illustrate the clinical relevance of this emerging ECG sign, to show that it may be present despite a transient classical STEMI pattern, and to demonstrate that it can also be associated with proximal LAD-D1 bifurcation critical disease within the context of multivessel coronary involvement. We further highlight the pathophysiological mechanism underlying SAFS and emphasise the need for broader recognition of mid-anterior/anterolateral STEMI equivalents/OMI in contemporary clinical practice.

## Case presentation

**Figure 1:**
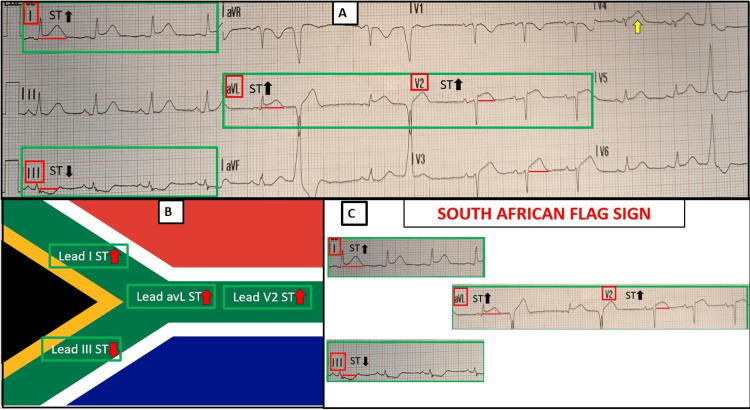
Initial ECG recorded during angina. (A) Sinus rhythm at a rate of 82 bpm with normal atrioventricular conduction. STE (measured at the J point) of 1 mm in the high lateral leads (I and aVL) and approximately 2 mm in V2 and V3 (distance from red line). A probable hyperacute T wave is present in V4 (yellow arrow), together with poor R-wave progression from V1 to V3. ST-segment depression is observed in lead III. Two single premature ventricular beats are noted. The ESC 2023 ACS STEMI criteria are met in the precordial leads (STE ≥ 1.5 mm in V2-V3 for females regardless of age) and in the anatomically contiguous leads I and aVL (STE ≥1 mm), although only at the borderline of significance. The meaning of the isolated contemporary ST elevation in the non-contiguous lead V2 is not addressed by the current ESC guidelines [5]. (B) and (C) South African flag sign: The pattern of STE in non-contiguous leads I-aVL-V2, accompanied by reciprocal ST deviation in lead III, constitutes the “South African flag sign,” a finding indicative of OMI usually due to the acute critical interruption of blood flow in the first diagonal branch of the LAD. ESC: European Society of Cardiology; ACS: acute coronary syndrome; STE: ST-segment elevation; STEMI: ST-segment elevation myocardial infarction; OMI: occlusion myocardial infarction; LAD: left anterior descending artery Source: Flag of South Africa (public domain), sourced from Wikimedia Commons (https://commons.wikimedia.org/wiki/File:Flag_of_South_Africa.svg). All image modifications were performed solely and exclusively for educational and illustrative purposes.

A woman in her 80s developed a sudden onset of severe, oppressive chest pain (8/10) radiating to her neck while performing light physical activity at home, a few hours before seeking medical attention. Her medical history was significant for arterial hypertension treated with valsartan and a suspected, though never formally investigated, glucose-6-phosphate dehydrogenase (G6PD) deficiency. She reported no drug allergies, no prior history of chest pain or dyspnea and no previous ECGs were available for comparison. The emergency physician obtained the first ECG within minutes (Figure 1A).

An ACS was suspected, and the ECG was transmitted via the wireless STEMI network to our coronary care unit for cardiology assessment. STE was identified in the non-contiguous leads I-aVL and V2, with reciprocal ST depression in lead III, consistent with the SAFS and suggesting occlusion of D1. Additional STE of approximately 1.8 mm was present in V3, together with a presumably hyperacute T wave (HTW) in V4, indicating possible anterior extension of the infarction. The emergency physician administered intravenous acetylsalicylic acid 300 mg and unfractionated heparin 5,000 IU and transferred the patient to our cardiology unit for emergent coronary angiography.

**Figure 2:**
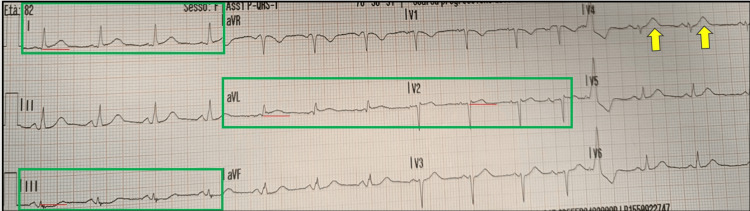
ECG recorded 20 minutes after the initial tracing demonstrating SAFS despite resolution of STEMI criteria. It shows regression of the anterior and lateral ST-segment elevation. The ESC ACS criteria were no longer fulfilled [6]. Nevertheless, a mild residual ST-segment elevation (<1 mm) persisted in leads I, aVL, and V2, with concomitant ST-segment depression in lead III (green boxes), consistent with the SAFS. This pattern represents a STEMI-equivalent and is indicative of OMI requiring an immediate reperfusion strategy. Coexisting HTW in lead V4 (yellow arrows) were again evident. ECG: electrocardiogram; ESC: European Society of Cardiology; ACS: acute coronary syndrome; SAFS: South African flag sign; STEMI: ST-elevation myocardial infarction; OMI: occlusion myocardial infarction; HTW: hyperacute T waves

A second ECG was recorded during transport (Figure 2). This tracing showed regression of the STE (overall duration < 20 minutes), which was very subtle and limited to leads I-aVL and V2, with minimal horizontal ST depression in lead III; a likely HTW in V4 persisted. STEMI criteria were no longer fulfilled [6]; however, the SAFS remained present.

Approximately 40 minutes after first medical contact, the patient arrived at our department, still symptomatic for angina and was transferred directly to the catheterisation laboratory. After reviewing the second ECG, the presence of a definite STEMI was considered uncertain, and a diagnosis of “very high-risk NSTEMI” was proposed due to refractory chest pain, transient STE and dynamic ST-T changes. The patient’s vital signs were within normal limits. Blood samples were obtained for routine laboratory testing, cardiac biomarkers, and measurement of G6PD activity, given the reported but unconfirmed history of G6PD deficiency. Informed consent was obtained, and coronary angiography was performed via the left radial artery.

**Figure 3:**
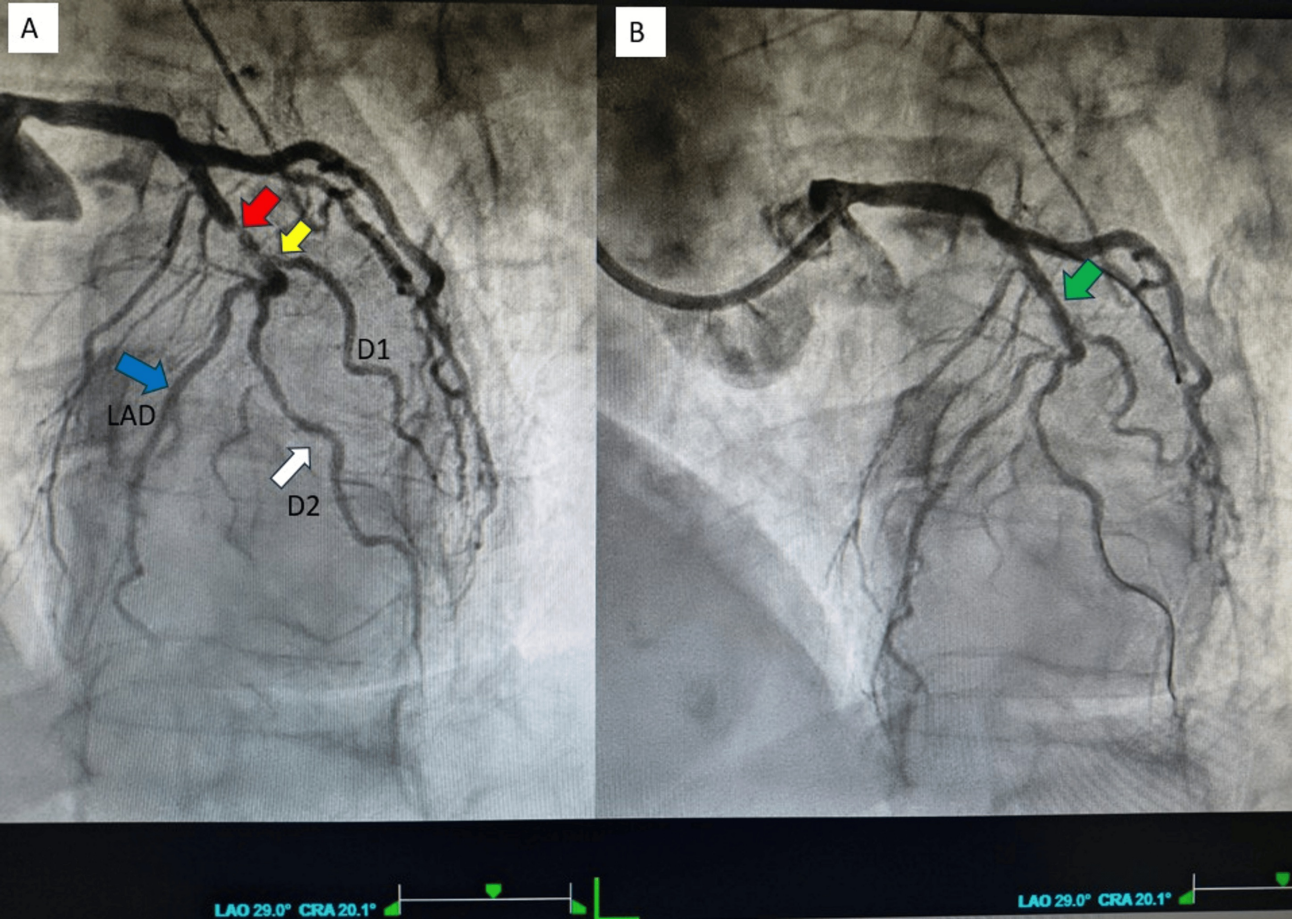
Left coronary angiography in LAO-CRA views. (A) A subocclusive lesion (red arrow) is identified at the proximal LAD-D1 bifurcation, beginning distal to the septal branches and proximal to D1. The lesion is partially recanalised and involves the ostium of D1 (yellow arrow), with reduced TIMI 1 flow. A well-developed D2 (white arrow) arises from the LAD and courses parallel to D1 to supply the anterolateral wall. The blue arrow highlights the course of the LAD towards the apex after giving rise to D2. (B) Recanalisation of the proximal LAD lesion (green arrow) after non-compliant balloon dilatation, prior to implantation of a drug-eluting stent. LAO: left anterior oblique; CRA: cranial; LAD: left anterior descending; D1: first diagonal branch; TIMI: thrombolysis in myocardial infarction; D2: second diagonal branch

Using 6F Judkins left (JL 4) and Judkins right (JR) catheters, coronary angiography demonstrated a sub-occlusive aterothrombotic lesion (culprit) in the proximal LAD artery (Figure 3A, red arrow), extending to the D1 ostium (Figure 3A, yellow arrow), with thrombolysis in myocardial infarction (TIMI) 1 flow (Video 1, left panel). A well-developed second diagonal branch (D2) was also present (Figure 3A, white arrow), with reduced flow due to the upstream stenosis. In addition, a mild stenosis was observed at the LAD - intermediate branch (ramus intermedius) - left circumflex (LCx) trifurcation, along with a critical stenosis of the mid right coronary artery (RCA). The proximal LAD lesion was predilated with a 2.0 mm balloon (Figure 3B and Video 1, right panel), followed by implantation of a 2.25 x 18 mm Onyx Frontier drug-eluting stent (Medtronic, Minneapolis, MN, USA), achieving a good final angiographic result. During the procedure, 2,000 IU of unfractionated heparin and a loading dose of ticagrelor 180 mg were administered.

**Video 1:** Video of left coronary angiography in LAO-CRA views. Left panel: Subocclusive lesion LAD-D1 bifurcation involving the ostium of D1, with reduced TIMI 1 flow. A well-developed D2 arises from the LAD and courses parallel to D1 to supply the anterolateral wall. Right panel: Recanalisation of the proximal LAD lesion after non-compliant balloon dilatation, prior to implantation of a drug-eluting stent. LAO: left anterior oblique; CRA: cranial; LAD: left anterior descending; D1: first diagonal branch; TIMI: thrombolysis in myocardial infarction; D2: second diagonal branch

**Figure 4:**
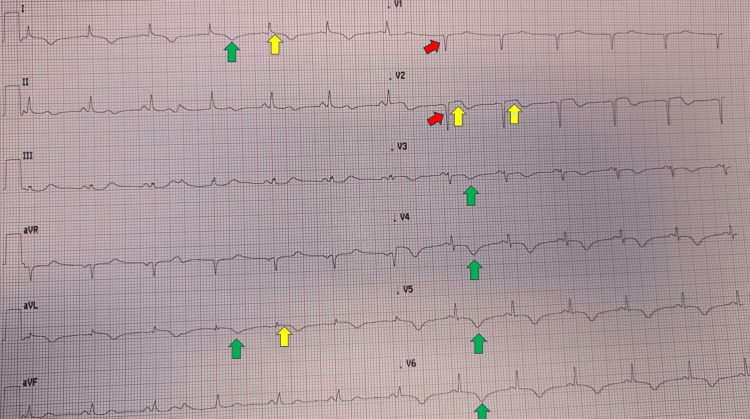
Electrocardiogram recorded on the day following the procedure. Sinus rhythm at a normal heart rate is present, with signs of septal necrosis (“qs pattern,” red arrows) and symmetric negative T waves in the anterolateral leads (green arrows). A mild residual ST-segment elevation (<1 mm, yellow arrows) persists in the SAFS leads I-avL and V2 with a positive-biphasic T wave, suggesting no-reflow due to microvascular obstruction injury. SAFS: South African flag sign

The ECG recorded on the following day (Figure 4) demonstrated anteroseptal necrosis (qs pattern, red arrows) with diffuse anterolateral reperfusion-associated repolarisation abnormalities (T-wave inversion, green arrows). A persistent subtle STE in lead I-avL-V2 was also observed (yellow arrows), consistent with possible no-reflow due to microvascular obstruction injury of the mid-anterior/anterolateral wall. Subsequent ECGs showed persistent antero-septal necrosis.

**Figure 5:**
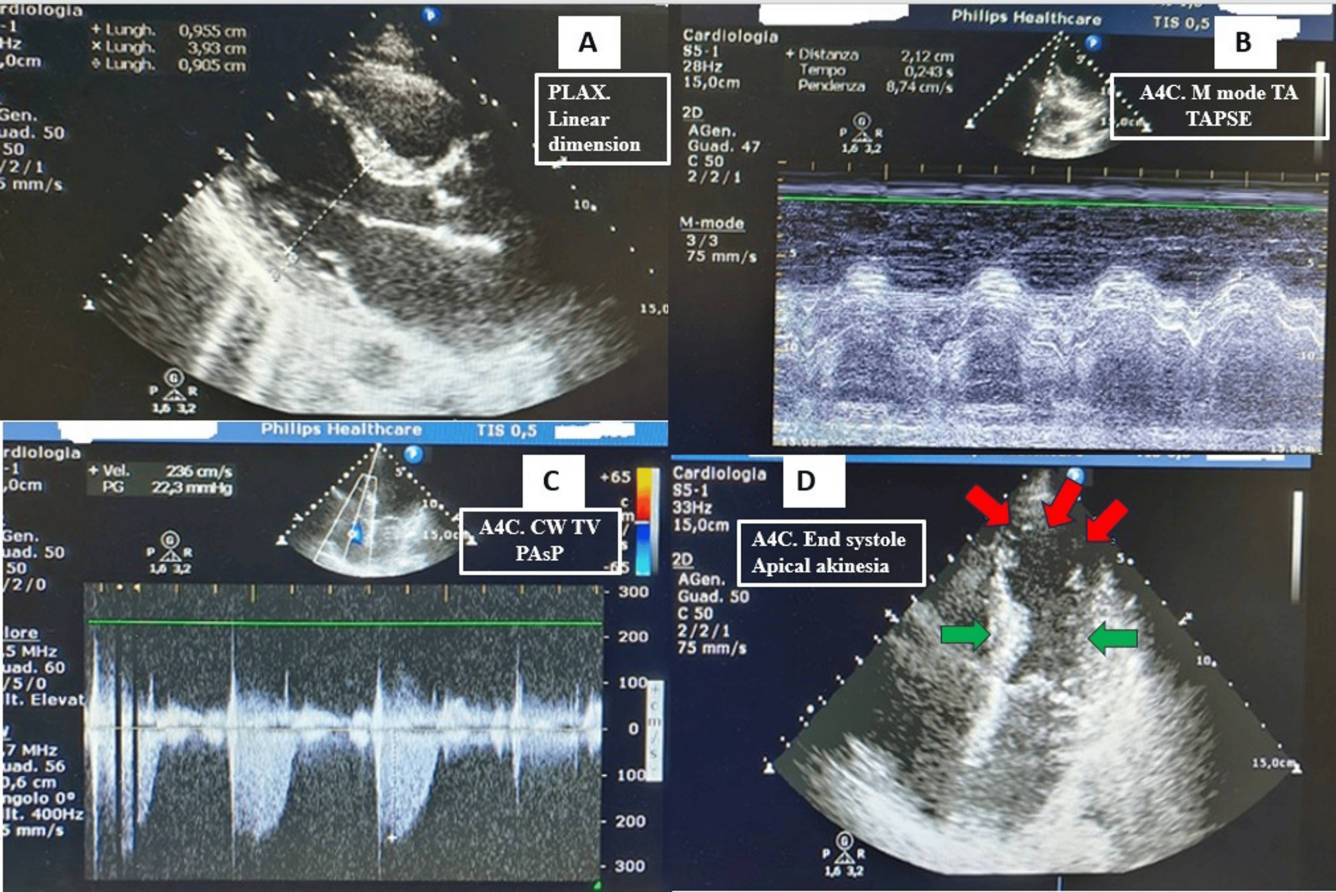
Echocardiography performed the day after angioplasty. (A) PLAX view showing normal wall thickness and normal intracavitary dimensions. (B) A4C M-mode at the TA demonstrating TAPSE within normal limits (21 mm). (C) A4C CW Doppler across the TV showing normal estimated PASP of 25 mmHg (22 mmHg + RAP 3 mmHg). (D) A4C end-systolic frame showing apical akinesia (red arrows) with preserved contraction of the basal segments (green arrows). PLAX: parasternal long-axis view; A4C: apical four-chamber view; TA: tricuspid annulus; TAPSE: tricuspid annular plane systolic excursion; CW: continuous wave; TV: tricuspid valve; PASP: pulmonary artery systolic pressure; RAP: right atrial pressure

The transthoracic echocardiogram performed the day after admission (Figure 5 and Video 2) showed normal chamber dimensions and wall thicknesses, apical akinesia with moderately reduced left ventricular systolic function (Simpson biplane ejection fraction 39%), and normal right ventricular size and function (tricuspid annular plane systolic excursion (TAPSE) 22 mm). There was mild mitral, aortic and tricuspid regurgitation. Estimated pulmonary artery systolic pressure was normal (25 mmHg), with no pericardial effusion and normal inferior vena cava size and collapsibility.

**Video 2:** Video of echocardiography performed the day after angioplasty. PLAX view shows normal basal segments' wall motion and mild aortic regurgitation. A4C and A2C views show apical akinesia, normal basal segments' wall motion and mild mitral regurgitation. PLAX: parasternal long-axis view; A4C: apical four-chamber view; A2C: apical two-chamber view

Table 1 reports laboratory values at admission and at discharge. Evidence of myocardial injury is noted, with a marked troponin peak on the second day and a significant increase in N-terminal pro-B-type natriuretic peptide (NT-proBNP). In addition, low-density lipoprotein cholesterol exceeds the recommended target of 55 mg/dL, whereas the measured G6PD activity is within the normal range, thereby excluding G6PD deficiency.

**Table 1:** Laboratory values at admission and at discharge. Peak troponin levels (on day 2) and NT-proBNP concentrations were 26,607 ng/L and 3,398 pg/L, respectively. WBC: white blood cell count; RBC: red blood cell count; Hb: hemoglobin; Ht: Plt: platelet count; hsTn-I: high-sensitivity cardiac troponin I; NT-proBNP: N-terminal pro-B-type natriuretic peptide; G6PD: Glucose-6-phosphate dehydrogenase; Cre: creatinine; Na+: sodium; K+: potassium; total-C: total cholesterol; HDL-C: high-density lipoprotein cholesterol; LDL-C: low-density lipoprotein cholesterol; TG: triglyceride; CRP: C-reactive protein; LDH: lactate dehydrogenase; CK: creatine kinase; AST: aspartate aminotransferase; ALT: alanine aminotransferase; TSH: thyroid-stimulating hormone

Value	Admission	Discharge	Reference range	Unit
WBC	16,000	8000	4-9.5	x10^3^/microL
RBC	4.8	4.7	4-5.5	x10^6^/microL
Hb	9	9.5	13-17	g/dL
Plt	250	260	150-350	x10^3^/microL
hsTn-I	2,771	53	<12	ng/L
NT-proBNP	750	1,035	<450	pg/mL
G6PD	15	-	>10.22	U/g Hb
Cre	0.95	1.1	0.6-1.1	mg/dL
Na+	135	137	135-145	mEq/L
K+	3.7	4.3	3.5-5	mEq/L
Glucose	190	98	60-100	mg/dL
Total-C	193	-	<200	mg/dL
HDL-C	42	-	40-90	mg/dL
LDL-C	137	-	<110	mg/dL
TG	126	-	<150	mg/dL
CRP	0.5	0.9	<1	mg/dL
LDH	300	150	<247	U/L
CK	280	120	<145	U/L
AST	98	48	<50	U/L
ALT	47	42	<50	U/L
Albumin	4	-	3.5-5.2	g/dL
TSH	0.68	-	0.4-4	U/L

**Figure 6:**
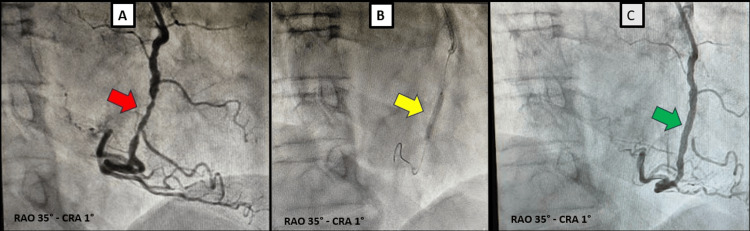
Right coronary artery angiography in RAO CRA projection. (A) Critical stenosis of the mid RCA (red arrow). (B) DES implantation at the site of the lesion (yellow arrow). (C) Optimal final angiographic result with restoration of normal coronary flow (green arrow). RAO: right anterior oblique; CRA: cranial; RCA: right coronary artery; DES: drug-eluting stent

On hospital day 4, another coronary angiography was performed to complete revascularisation of the RCA. A critical mid-RCA stenosis was confirmed (Figure 6A and Video 3, left panel). After crossing the lesion, predilatation was performed with a non-compliant balloon, followed by implantation of a drug-eluting stent (Figure 6B), achieving an optimal final angiographic result (Figure 6C and Video 3 right panel) without procedural complications.

**Video 3:** Video of right coronary artery angiography in RAO CRA view. Left panel: Critical stenosis of the mid RCA. Right panel: Optimal final angiographic result after DES implantation with restoration of normal coronary flow. RCA: right coronary artery; RAO: right anterior oblique; CRA: cranial; DES: drug-eluting stent

During hospitalisation, the patient received dual antiplatelet therapy (DAPT) (acetylsalicylic acid 100 mg plus ticagrelor 90 mg twice daily), ezetimibe/atorvastatin 10/40 mg, bisoprolol 2.5 mg, furosemide 25 mg twice daily, valsartan 40 mg twice daily, potassium canrenoate and amoxicillin/clavulanate for neutrophilic leukocytosis associated with lower urinary tract infection. The clinical course was uneventful, with no arrhythmias or episodes of acute heart failure, and the patient remained free of angina.

She was discharged after 13 days with a diagnosis of anterior STEMI and an indication for at least 12 months of DAPT, to be prolonged with reduced-dose ticagrelor if well tolerated, along with lipid-lowering therapy (statin + ezetimibe), and anti-heart failure therapy for moderate left ventricular dysfunction.

## Discussion

STEMI criteria are not perfect in identifying complete coronary occlusions, being affected both by false negatives, with potentially dramatic consequences, and by false positives, which are time- and resource-consuming [1,6]. Moreover, they may be difficult to apply in electrocardiographic settings not specifically addressed by current guidelines and may fail to identify the culprit artery in complex coronary scenarios [5,7]. Although the specific electrocardiographic features were originally described by Sclarovsky et al. and linked to mid-anterior/anterolateral myocardial infarction, the SAFS has only recently been codified and is increasingly recognised as an ECG pattern of OMI that warrants immediate reperfusion therapy [2,3,6].

In 2015, Durant and Singh reported a case describing a 93-year-old patient without assigning a specific name to the pattern [8]. The following year, Littmann proposed it as a teaching tool to improve recognition of high-lateral STEMI [4].

Over the subsequent decade, and particularly during the past year, an increasing number of patients exhibiting this sign have been reported in the literature. The culprit vessel is most commonly D1, but the pattern may also be produced by lesions involving the LAD-D1 bifurcation, the intermediate branch, or the second diagonal branch [2,9-11].

The acute total occlusion of the circumflex artery or obtuse marginal branch does not typically produce the SAFS; instead, it usually causes STE in the lateral leads, possibly associated with inferior leads, or presents as a posterior STEMI (maximal ST-segment depression in V1-V4) [6,12].

Our case involves a more complex coronary anatomy than those previously reported. Specifically, it includes an intermediate branch that is not the culprit lesion; the first diagonal branch, which is not the sole culprit, as it is affected by a critical ostial lesion involving the bifurcation; and the second diagonal branch, which demonstrates reduced blood flow secondary to a proximal LAD-D1 bifurcation stenosis.

For these reasons, SAFS should not be considered a specific sign of D1 occlusion, but rather a marker of mid-anterior/anterolateral infarct localisation, in which the culprit lesion may involve the LAD-D1 bifurcation or a vessel (intermediate branch or second diagonal branch) supplying that territory.

In our case, SAFS is present on both ECGs, but is more clearly evident in Figure 1, together with the STEMI criteria, also involving the anterior leads. In contrast, the ECG in Figure 2 shows regression of STE due to likely partial recanalisation of the lesion, with SAFS remaining as the sole sign of OMI. In addition, the presence of STE in V3 in Figure 1 is consistent with Birnbaum’s description, which, in the setting of concomitant STE in aVL and V2, demonstrated a positive predictive value of 95% and a negative predictive value of 94% for the LAD (and not D1) as the infarct-related artery [13].

Although rarely, critical multivessel disease unmasked by SAFS had previously been reported in association with LCx and posterolateral artery disease, but not involving the RCA [11,14,15]. Our case confirms that this pattern may reveal multivessel coronary artery disease and, for the first time, involvement of the RCA.

Even in earlier descriptions, the myocardial territory supplied by the culprit vessel was limited to the high-lateral [4], mid-anterior, and mid-lateral regions [3]. However, myocardial injury may be more extensive and lead to ventricular dysfunction and reduced ejection fraction, as observed in our case. This is likely to occur more frequently when the responsible lesion involves the LAD-D1 bifurcation or when concomitant multivessel coronary artery disease is present, resulting in a larger amount of myocardium at risk [9,14].

Our case demonstrates that SAFS is not necessarily an isolated finding. It may occur after a transient STEMI, likely reflecting partial spontaneous thrombus recanalisation, and may result from proximal LAD thrombosis extending from the bifurcation to D1. In addition, SAFS may indicate underlying critical multivessel coronary artery disease, including RCA involvement, and can be associated with extensive myocardial injury and reduced left ventricular ejection fraction. Importantly, SAFS should not be confused with NSTEMI, as it differs in both prognosis and urgency of percutaneous revascularisation.

## Conclusions

This case illustrates that the SAFS remains a simple and rapidly recognisable electrocardiographic marker of OMI, whose broader awareness may help prevent delayed diagnosis and adverse outcomes. Although traditionally associated with critical stenosis or occlusion of the first diagonal branch, SAFS should not be regarded as highly specific for this pattern alone. As demonstrated in our case, it may also reflect thrombotic occlusion of the proximal LAD coronary artery, particularly when the bifurcation with the first diagonal branch is involved, and may even signal the presence of underlying multivessel coronary artery disease. This pattern may also be associated with extensive myocardial damage and reduced ejection fraction.

Recognising SAFS as a dynamic ECG manifestation of ACO, regardless of the presence or absence of classical contiguous STE, is therefore essential. Its identification should prompt urgent reperfusion strategies and prevent misclassification as NSTEMI, reducing delays in revascularisation, ultimately improving clinical decision-making and patient prognosis. Prospective studies are required to validate this pattern, confirm these observations, and clarify their diagnostic and management implications across the spectrum of patients presenting with this electrocardiographic sign.
